# Individual Differences in Frequency and Saliency of Speech-Accompanying Gestures: The Role of Cognitive Abilities and Empathy

**DOI:** 10.1037/a0033861

**Published:** 2013-08-05

**Authors:** Mingyuan Chu, Antje Meyer, Lucy Foulkes, Sotaro Kita

**Affiliations:** 1 Neurobiology of Language Department, Max Planck Institute for Psycholinguistics, Nijmegen, the Netherlands; 2 Psychology of Language Department, Max Planck Institute for Psycholinguistics, and School of Psychology, Radboud University, Nijmegen, the Netherlands; 3 Department of Clinical, Educational and Health Psychology, University College London, London, England; 4 School of Psychology, University of Birmingham, Birmingham, England

**Keywords:** gesture production, working memory, spatial transformation ability, conceptualization ability, empathy

## Abstract

The present study concerns individual differences in gesture production. We used correlational and multiple regression analyses to examine the relationship between individuals’ cognitive abilities and empathy levels and their gesture frequency and saliency. We chose predictor variables according to experimental evidence of the functions of gesture in speech production and communication. We examined 3 types of gestures: representational gestures, conduit gestures, and palm-revealing gestures. Higher frequency of representational gestures was related to poorer visual and spatial working memory, spatial transformation ability, and conceptualization ability; higher frequency of conduit gestures was related to poorer visual working memory, conceptualization ability, and higher levels of empathy; and higher frequency of palm-revealing gestures was related to higher levels of empathy. The saliency of all gestures was positively related to level of empathy. These results demonstrate that cognitive abilities and empathy levels are related to individual differences in gesture frequency and saliency.

People spontaneously produce gestures when they speak. Gestures occur across cultures (Kendon, 2004; Kita, 2009), ages (Feyereisen & de Lannoy, 1991), and communicative contexts (McNeill, 1992). Children start gesturing at the one-word stage of language development (Iverson & Goldin-Meadow, 2005). Congenitally blind speakers gesture when talking to blind listeners (Iverson & Goldin-Meadow, 1998). However, gesturing is not a mandatory element of speaking or communication, and people differ substantially in the frequency and saliency of their gestures. The present study investigated which factors contribute to such individual differences. We correlated the frequency and saliency of speakers’ gestures with scores reflecting their level of empathy and performance on a range of cognitive tasks, and determined how well each of these scores predicted gesture frequency and saliency.

Several earlier studies have reported group differences in gesturing. For instance, when asked to describe complex objects, young women produced more descriptive gestures (i.e., gestures that carry the semantic meaning of a verbal message) than elderly women, although these two age groups produced nondescriptive gestures equally often (Cohen & Borsoi, 1996). Women produced more gestures than men when asked to describe an animated cartoon (Hostetter & Hopkins, 2002). Italian speakers gestured more often than British English speakers (Graham & Argyle, 1975). However, these studies did not elucidate the psychological processes underlying the group differences.

There are a few correlational studies similar to our study that have aimed to understand within-group individual differences in gesturing. For instance, in an interview, people who often expressed negative affect produced more descriptive gestures than people who did so less often (Wiens, Harper, & Matarazzo, 1980). When speakers were asked to define English words, such as *tornado* and *hesitation*, their frequency of representational gestures (i.e., gestures that depict or indicate an entity by the hand shape and movement trajectories) was positively correlated with their level of extraversion (i.e., energy, gregariousness, and sociability) and neuroticism (i.e., excessive rumination, low self-esteem, and shifting self-concepts; Hostetter & Potthoff, 2012). When describing solutions of geometric analogy problems, people with high fluid intelligence produced more representational gestures than people with average fluid intelligence (Sassenberg, Foth, Wartenburger, & van der Meer, 2011). Finally, participants were asked to describe a short cartoon video and explain how to wrap a package, and were divided into nine groups based on their verbal skill (low, average, high) and spatial skill (low, average, high; Hostetter & Alibali, 2007). In this study, people with low verbal skill but high spatial skill gestured more often than any of the other eight groups.

The current study extends the earlier work in a number of ways. First, we chose predictor variables on the basis of existing experimental evidence concerning the functions of gesture. This is because the experimental investigations suggest links between gesture production and certain cognitive processes that can also manifest themselves in a correlational study (e.g., Underwood, 1975). For instance, if experimental evidence suggests that gesturing supports spatial transformation, one might expect that people with weak spatial transformation ability would produce more gestures to help them transform spatial concepts when they speak, compared to people with stronger spatial transformation ability. Second, whereas most of the earlier studies assessed rather homogeneous groups of participants, we included high school students and young people in vocational training as well as undergraduate students. This allowed us to assess participants with a wide range of cognitive abilities. Finally, whereas most of the earlier correlational studies only concerned the frequency of representational gestures, we included conduit and palm-revealing gestures as well and examined not only the frequency but also the saliency of the gestures. In the following sections, we first explain how we defined gesture type and saliency, and then explain how we chose our predictor variables.

## Defining Gesture Types and Saliency

We used two gesture elicitation tasks to elicit gestures, one requiring participants to define phrases, such as *to intervene*, and one requiring them to solve social dilemmas. These tasks are good approximations of everyday verbal tasks. They neither encourage nor discourage the use of gestures, which ensures high variability in gesture frequency.

Our gesture categorization followed conventions from earlier research, especially McNeill (1992) and Bavelas, Chovil, Lawrie, and Wade (1992). We distinguished three types of gestures: representational, conduit, and palm-revealing gestures. Distinguishing these different types of gestures is important because they may serve different functions, so they may be related to different social and cognitive skills. *Representational gestures* depict a concrete or abstract concept with the shape or motion of the hands (*iconic gestures* and *metaphoric gestures* in McNeill, 1992), or point to a referent in the physical or imaginary space (*concrete or abstract deictic gestures* in McNeill, 1992). In *conduit gestures*, the palm of the hand faces upward and moves toward the listener as if to present a clearly formulated idea on the palm to the listener (*conduit metaphor gesture* in McNeill, 1992; a type of *interactive gesture* in Bavelas et al., 1992). Conduit gestures are similar to representational gestures in that both are used to present the speaker’s idea to the listener, but conduit gestures additionally manage interaction by bringing the listener into the conversation. In *palm-revealing gestures*, the palm is revealed to the listener as if to indicate uncertainty or having nothing to say (another type of interactive gesture in Bavelas et al., 1992) by showing an empty hand. They are sometimes accompanied by a shoulder shrug. Unlike conduit gestures, palm-revealing gestures manage interaction without representing any content on the palm. In this sense, palm-revealing gestures are purely interactive.

Whereas most individual differences studies only measured gesture frequency, we also measured gesture saliency. We did so by measuring the size (the body part used for a gesture: fingers, hand, forearm, or whole arm) and height (highest point of a gesture: below waist, between waist and chin, and above chin) of the gesture. Thus, the most salient gesture would be produced with the whole arm and above the chin; the least salient one would be produced with fingers below the waist.

## Choice of Predictor Variables

In this section we explain how we derived the predictor variables from earlier experimental studies concerning the functions of gestures. It should be noted that gestures may serve multiple functions simultaneously (e.g., Alibali, Heath, & Myers, 2001; Jacobs & Garnham, 2007). As the present study is correlational, its results cannot test the functions of gesture. To do so, one has to experimentally manipulate the gesture production (e.g., by encouraging or prohibiting gesture) and observe the effect on speech production and communication. However, previous experimental investigations on the functions of gesture suggest links between gesture production and certain cognitive processes, which can help us to formulate hypotheses concerning the expected correlations.

### Empathy

It has often been proposed that gestures facilitate communication between the speaker and the listener (e.g., Bavelas, 1994; Bavelas & Chovil, 2000; Clark, 1996; Kendon, 2004). This hypothesis is supported by studies showing that speakers produce gestures more often in face-to-face communication, compared to when the listener is out of sight (Alibali et al., 2001; Krauss, Dushay, Chen, & Rauscher, 1995). Furthermore, speakers produce larger and more precise gestures when they describe new information to their listener than when they describe old information (size, Holler & Stevens, 2007; precision, Gerwing & Bavelas, 2004) and when they are more motivated to communicate clearly than when they are less motivated (size, Hostetter, Alibali, & Schrager, 2011; precision, Gullberg, 2006). In sum, speakers use gestures to communicate effectively and to support their listener.

If gestures are used to support communication, speakers who care more about the quality of their communication may gesture more frequently and/or more saliently than speakers who care less. Caring about the quality of communication might be related to *empathy*, the degree to which one recognizes and understands other people’s thoughts and feelings. Specifically, individuals who are more empathic may be more motivated to communicate clearly to their partner. The link between empathy and gesture frequency is supported by studies showing that individuals with autism, who have lower levels of empathy (Baron-Cohen & Wheelwright, 2004), produce fewer gestures than controls (Buffington, Krantz, McClannahan, & Poulson, 1998; but see de Marchena & Eigsti, 2010, for conflicting findings). To assess empathy level, we used the Empathy Quotient questionnaire developed by Baron-Cohen and Wheelwright (2004). We expected that speakers with higher levels of empathy would gesture more frequently and more saliently than participants with lower levels of empathy. This should be the case for all three types of gestures.

### Working Memory Capacity

Gestures are produced not only to support listeners’ comprehension, but also to lighten the working memory load for speakers (e.g., de Ruiter, 1988, 2000; Goldin-Meadow, 2003; Wagner, Nusbaum, & Goldin-Meadow, 2004). It has been proposed that representational gestures can help speakers to maintain mental images in visuospatial working memory by boosting the activation level of the mental images (de Ruiter, 1988, 2000). In support of this proposal, de Ruiter (1998) and Wesp, Hesse, Keutmann, and Wheaton (2001) demonstrated that speakers produced representational gestures more often when they described pictures from memory than when they could see the pictures during their description. With respect to alleviating visuospatial working memory load, conduit gestures may have a similar function for speakers as representational gestures. Conduit gestures overtly refer to concepts and place them in particular spatial locations. This may increase their activation level and support their maintenance in visuospatial working memory.

In addition to visuospatial working memory load, representational gestures have been hypothesized to lighten speakers’ verbal working memory load (Goldin-Meadow, 2003; Wagner et al., 2004). In Wagner et al. (2004), adult participants were asked to explain solutions of math equations while simultaneously carrying out a verbal working memory task (memorizing a string of letters) or a visual working memory task (memorizing a pattern of black squares in a grid). When participants were allowed to gesture, they performed better on both working memory tasks than when they were prohibited from gesturing. Ping and Goldin-Meadow (2010) showed that children’s performance on both the verbal and visual working memory tasks was improved by gesture production. They concluded that representational gestures could lighten working memory load during speech production, regardless of the type of information to be stored.

As representational and possibly conduit gestures reduce speakers’ visuospatial and verbal working memory load, we predicted that speakers with relatively poor visuospatial or verbal working memory capacity would produce representational and conduit gesture more often than speakers with better working memory capacity. To assess working memory capacity, we tested participants on three working memory tasks, measuring verbal (digit span task; Wechsler, 1939), visual (visual pattern test; Della Sala, Gray, Baddeley, & Wilson, 1997), and spatial (Corsi block task; Corsi, 1972) working memory capacity. Visual working memory (i.e., the ability to keep static visual images in working memory) and spatial working memory (i.e., the ability to keep movements of visual images in working memory) were measured separately, as these are dissociable components of working memory shown by selective interference from either a visual or spatial secondary task, respectively (Della Sala, Gray, Baddeley, Allamano, & Wilson, 1999). We expected that speakers scoring low on visual, spatial, or verbal working memory tasks would produce representational and conduit gestures more often than speakers scoring higher on these tasks.

### Spatial Transformation and Conceptualization Abilities

Kita (2000) and Alibali, Kita, and Young (2000) proposed that representational gestures support the generation of conceptual units suitable for speaking. Expressing thoughts by speech requires speakers to linearize complex information and to focus on one chunk of information suitable for expression at a time (Levelt, 1989). Representational gestures may facilitate this process. Two processes are involved in this facilitation: manipulation of spatiomotoric information and segmenting out spatiomotoric information into units suitable for speaking. First, mental images sometimes need to be transformed for verbalization. For instance, when describing a route, the speaker may need to transform mental images to match the listener’s perspective (e.g., Emmorey, Tversky, & Taylor, 2000; Levelt, 1989). In line with the view that representational gestures may facilitate such transformations, Chu and Kita (2011) found that participants produced representational gestures more frequently when solving difficult mental rotation problems than when solving easy ones. In addition, they observed that encouraging participants to gesture enhanced mental rotation performance.

**Figure 1 fig1:**
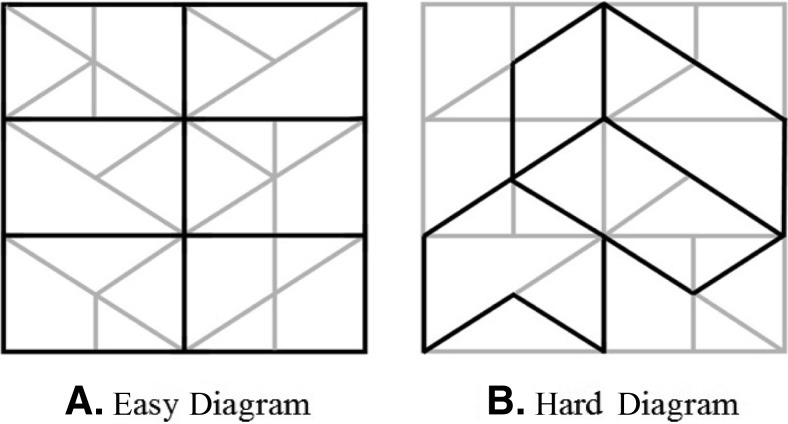
Two of the diagrams used in the conceptualization task.

The second process that representational gestures may facilitate is the segmentation of relevant spatiomotoric information into conceptual units suitable for speaking (Kita, 2000). People produce representational gestures more frequently when it is difficult to select a suitable conceptual unit for speaking than when it is easier (Hostetter, Alibali & Kita, 2007; Kita & Davies, 2009; Melinger & Kita, 2007). In Kita and Davies (2009), participants were asked to describe a set of lines. They produced more representational gestures when those lines were superimposed with dark lines that created distracting shapes than when all lines had the same color (see Figure 1). Kita and Davies proposed that the distracting lines made the images harder to conceptualize and to break down into suitable units for speaking and that representational gestures helped participants overcome this difficulty. With respect to segmentation, we expected that conduit gestures might again be functionally similar to representational gestures, as they highlight and externalize particular pieces of information and break down information into units suitable for speaking.[Fig-anchor fig1]

In the present study we used the mental rotation task from Chu and Kita (2011) to measure spatial transformation ability and the conceptualization task from Kita and Davies (2009) to measure conceptualization ability. We expected that speakers who performed poorly on one or both of these tasks would produce more representational and conduit gestures than those who performed better on these tasks.

### Lexical Retrieval Ability

It has been hypothesized that representational gestures serve as cross-modal primes and support lexical access during speech production (Krauss, Chen, & Chawla, 1996; Rauscher, Krauss, & Chen, 1996). The first line of evidence for this hypothesis comes from a study that examined the effect of lexical retrieval difficulty on the frequency of representational gestures. In a cartoon description task, participants produced representational gestures more frequently when they were instructed either to use as many obscure words as possible or to avoid using words that contained a specified letter than when there were no such restrictions (Rauscher et al., 1996). The second line of evidence for this hypothesis comes from studies that examined the effect of gesture prohibition on lexical retrieval. Participants spoke more slowly and produced more dysfluencies when gesturing was prohibited than when it was allowed (Rauscher et al., 1996). When presented with definitions of low-frequency words and asked to retrieve the target words, participants in a gesture-allowed condition retrieved more words and resolved more tip-of-the-tongue states (i.e., states where known words are temporarily inaccessible) than participants in a gesture-prohibited condition (Frick-Horbury & Guttentag, 1998; but see Beattie & Coughlan, 1998, for conflicting findings).

We assessed lexical retrieval ability through picture naming and name–picture verification tasks. We expected that speakers with poor lexical retrieval ability would produce representational gesture more often than speakers with better lexical retrieval ability.

## Summary of the Study

The present study concerns individual differences in gesture frequency and saliency. We used a phrase definition task and a social dilemma task to elicit gestures. The dependent variables were the frequency and saliency of three types of gestures: representational gestures, conduit gestures, and palm-revealing gestures. Participants were tested on a range of cognitive tasks, chosen according to existing evidence concerning the functions of gestures. These tasks assessed verbal, visual, and spatial working memory capacity, spatial transformation ability, conceptualization ability, and lexical retrieval ability. Participants were not allowed to gesture in the tasks that measured these six cognitive abilities. This ensured pure measurement of individuals’ cognitive abilities without any influence from gesture production. We also measured participants’ level of empathy. We used correlational analysis and multiple regression analysis to determine the relative impact of each of the predictor variables.

Note that we predicted *negative* correlations between cognitive abilities and the frequency of representational and conduit gestures. For example, we expected that speakers with poor visual working memory capacity would produce representational and conduit gestures more often than speakers with better visual working memory capacity. This is because these gestures are hypothesized to support the maintenance of mental images in visual working memory. They should therefore be used most often by speakers who have relatively weak visual working memory capacity. The experimental evidence reviewed above is consistent with this view. However, one might also consider the opposite prediction, namely that there should be *positive* correlations between cognitive abilities and the frequency of representational and conduit gestures. This could be the case if habitually producing gestures improves certain cognitive abilities such that when the cognitive abilities are tested (even when gesturing is not allowed), participants who habitually produce more gestures perform better. We know of no evidence supporting this suggestion, but the design of our study allows us to examine the directions of the relationships between cognitive abilities and the frequencies of different types of gestures.

## Method

### Participants

The participants were 129 native British English speakers (76 female, 53 male). They were recruited from academically focused senior schools preparing for university, vocationally focused schools, and universities in the West Midlands area of the United Kingdom. The diversity of academic backgrounds ensured large individual differences in cognitive abilities and thus sufficient variance in the predictor variables. Seven participants were excluded because they failed to understand most tasks (one participant) or did not complete the study (six participants). The final sample consisted of 122 participants (71 female, 51 male) with an mean age of 19.41 years (*SD* = 4.85).

### Tasks

#### Gesture elicitation tasks

The following two tasks were used to measure participants’ gesture frequency and saliency. In the English phrase definition task (adapted from Kita, de Condappa, & Mohr, 2007), participants were asked to define the abstract meanings of eight written English phrases (e.g., *to intervene*, *to disclose something confidential*) without using concrete examples. In the social-dilemma-solving task, participants were first asked to silently read a social dilemma story (e.g., a person received two invitations and could not decide which one to accept) and then asked to explain how they would deal with such a situation, why they would do so, and what they thought other characters in the story would feel about their decision. There were three social dilemma stories. The written story stayed on the computer screen until participants finished their description. There was no time limitation to read or discuss the dilemma. The order of the two gesture elicitation tasks was counterbalanced across participants. The order of the items within each task was fixed.

Gesture was not mentioned in the instructions for these tasks. The experimenter sat opposite the participant and maintained eye contact throughout the description, but did not provide any verbal or nonverbal feedback. The computer screen was located on a table to the right of the participant.

#### Digit span task

The digit span task, adapted from Wechsler (1939), measured verbal working memory ability. A sequence of digits (e.g., 8, 3, 4, 1, 6) was presented on a computer screen at the rate of one digit per second. Each digit appeared only once within a sequence. After the end of the sequence, participants were asked to recall the digits in the order they had appeared. There were two practice trials (a three-digit and a four-digit sequence), after which the trials progressively increased in difficulty from five-digit to nine-digit sequences. There were 25 trials, with five trials at each difficulty level. The experimenter stopped the test when a participant failed on all five trials of a difficulty level. The participant’s score was the proportion of items correctly recalled.

#### Visual pattern task

**Figure 2 fig2:**
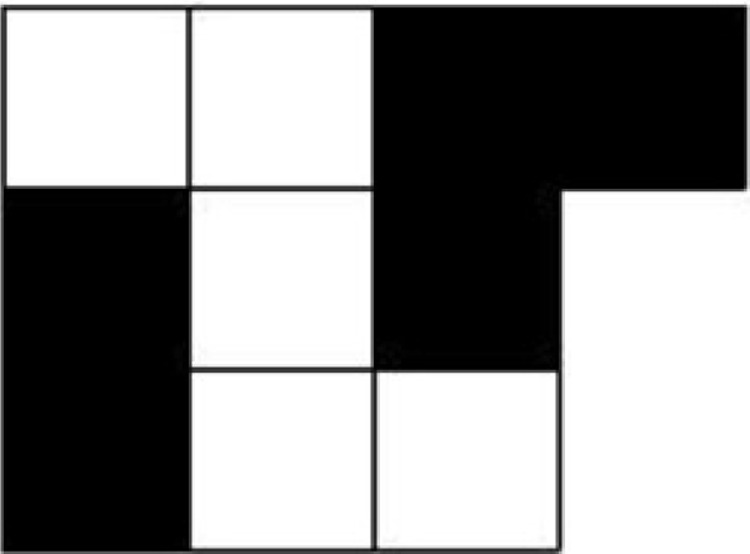
One of the stimuli used in the visual pattern task.

The visual pattern task, adapted from Della Sala et al. (1997), measured the visual component of visuospatial memory. Participants were shown grids in which half the cells were colored black, in various patterns (see Figure 2). The size of each cell was 15 mm × 15 mm. Each pattern was presented for 3 s and was then replaced by an all-white grid featuring a letter in each cell. Participants were asked to recall the pattern of the black cells by reading out the corresponding letters. Configurations that formed recognizable patterns were avoided. There were two practice trials with two and three black cells, respectively, after which the trials progressively increased in difficulty from seven to 11 filled cells. There were 25 trials in total, with five trials at each difficulty level. The experimenter stopped the test when a participant failed on all five trials of a difficulty level. The participant’s score was the proportion of correctly recalled items.[Fig-anchor fig2]

#### Corsi block task

**Figure 3 fig3:**
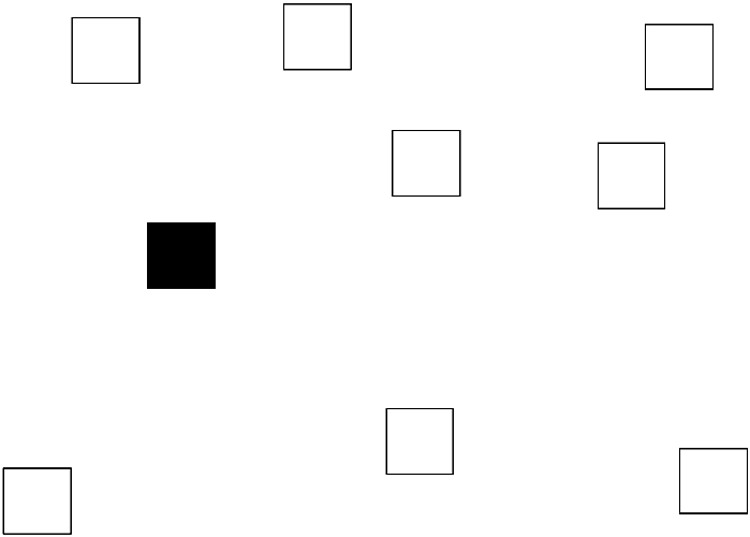
One of the stimuli used in the Corsi block task.

Whereas the visual pattern task assesses people’s ability to recall static visual details from working memory, the Corsi block task (adapted from Corsi, 1972) measured the spatial-sequential component of visuospatial working memory. In this task, nine blocks were irregularly placed on a white background on the computer screen (see Figure 3). The size of each block was 15 mm × 15 mm. One block at a time turned black for 1 s, with an interblock time of 0.5 s. Immediately after the end of the sequence, all nine blocks were filled with letters, and participants were asked to recall the sequence in which the black blocks had appeared by reading aloud the corresponding letters in order. There were two practice trials, featuring two and three blocks, respectively, after which the trials progressively increased in difficulty from five-block to eight-block sequences. There were 20 trials in total, with five trials at each difficulty level. The experimenter stopped the test when a participant failed at all five trials of a difficulty level. The participant’s score was the proportion of block sequences correctly recalled.[Fig-anchor fig3]

#### Mental rotation task

**Figure 4 fig4:**
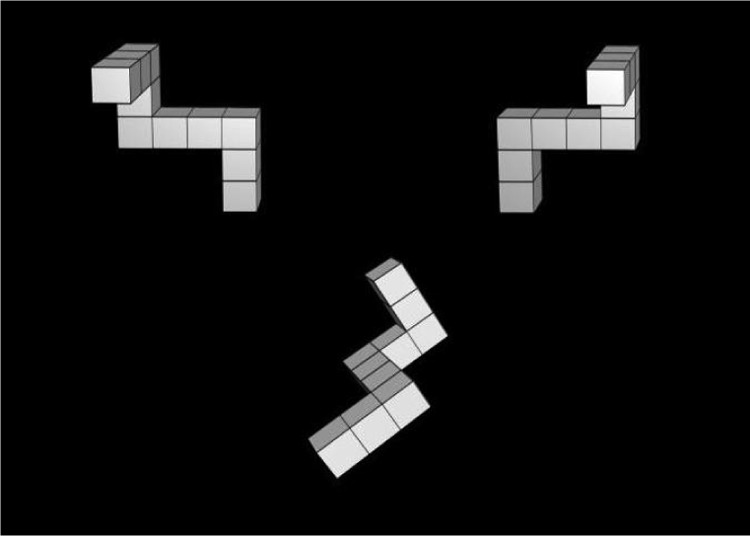
One of the stimuli used in the mental rotation task.

The mental rotation task, adapted from Shepard and Metzler (1971), measured spatial transformation ability. Three three-dimensional figures were presented on the computer screen (see Figure 4). The upper left and upper right objects were mirror images of each other. The lower object was a rotated version (derived through a rotation by 60°, 120°, 240°, or 300° around the bisector of two axes) of one of the two upper figures. The lower object corresponded to the upper left and upper right object on half the trials each. Participants were asked to decide as quickly and accurately as possible whether the lower object was a rotated version of the upper left or the upper right object by pressing one of two keys on the keyboard (*z* or *m*). There were two practice trials and 24 test trials. The participant’s score was the mean reaction time for correct responses.[Fig-anchor fig4]

#### Conceptualization task

The conceptualization task, adapted from Kita and Davies (2009), measured conceptual planning ability, in particular, the ability to select the most relevant conceptual units for speaking. Participants viewed four diagrams on the computer screen, one at a time. Each diagram consisted of six boxes, with each box containing horizontal, vertical, and diagonal lines. Participants were asked to describe the lines within each of the six boxes and to ignore the distinction between dark and light lines. In two diagrams (the easy condition), the boundaries of each box were highlighted with dark lines (see Figure 1A). In the remaining two diagrams (the hard condition), various geometric figures that spanned across multiple boxes were foregrounded by dark lines (see Figure 1B), which created Gestalt shapes irrelevant to the task and distracted speakers from conceptualizing lines in an optimal way. There was no time limitation to describe the lines. Each diagram remained on the computer screen until participants had completed their description. The order of the four trials was fixed.

Participants’ verbal descriptions were audio-recorded. The score of this task was the mean number of conceptual errors per box. Conceptual errors was counted if a participant described a line that spanned two boxes, described a straight line as two separate segments (e.g., the diagonal line in the top left rectangle in the right panel of Figure 1B), or described a line on the edge of a box (not required by the task, but highlighted in some figures such as Figure 1A). Speech errors—for instance, saying “left” instead of “right” or “vertical” instead of “horizontal”—were not counted as conceptual errors because it is not clear whether they were conceptual errors or lexical retrieval errors.

#### Picture naming task

The picture naming task was adapted from Snodgrass and Vanderwart (1980). Participants first saw a fixation cross for 1,000 ms, followed by a 100-ms blank screen and then a black-and-white line drawing of an object (e.g., a flag) for 4,000 ms. They were asked to name the object as quickly and accurately as possible. Their speech triggered a voice key, which recorded their response times. If a participant did not respond within 4,000 ms after picture onset, the trial terminated and the next trial started. There were four practice items and 62 experimental items, taken from Snodgrass and Vanderwart. The mean age of acquisition of the items was 1.97 years, the mean frequency was 2.90 per million words, and the mean length was 1.69 syllables. The mean naming latency from the correct trials was used to calculate the lexical retrieval ability.

In order to name an object, a speaker must identify the object first. Therefore, the picture naming latency consists of both the object recognition and the lexical retrieval latency. To purely estimate the lexical retrieval latency, we computed the difference between the picture naming latency and the latency of a name–picture verification task, which only required object recognition but not lexical retrieval (see below).

#### Name–picture verification task

The name–picture verification task was used together with the picture naming task to measure lexical retrieval ability. The name–picture verification task was adapted from Stadthagen-Gonzalez, Damian, Pérez, Bowers, and Marín (2009; see also Jescheniak & Levelt, 1994). Participants saw a word (e.g., *egg*) for 1,000 ms, followed by a blank screen for 100 ms and then by a black-and-white line drawing of an object (e.g., an egg or a bicycle pump) for 1,500 ms. Participants were asked to decide as quickly and accurately as possible whether the word matched the name of the drawing by pressing one of two keys on the keyboard. If the participant did not respond within 1,500 ms after picture onset, the trial was terminated and the next trial started. There were eight practice trials and 124 experimental trials. Half the trials were match trials, and the other half were mismatch trials. The items were taken from Snodgrass and Vanderwart (1980) and were not used in the picture naming task. The mean age of acquisition of the items was 1.87 years, the mean frequency was 2.90 per million words, and mean length was 1.62 syllables.

Jescheniak and Levelt (1994) argued that the latencies from mismatch trials should be used to indicate the name–picture verification latencies, as there might be lexical priming on match trials. In contrast, Stadthagen-Gonzalez et al. (2009) argued for the use of latencies from match trials because in their study visual and conceptual factors that are crucial for object recognition only correlated with latencies of match trials but not with latencies of mismatch trials. In our study, latencies on match and mismatch trials were highly correlated, *r*(122) = .89, *p* < .01. Therefore, we used the mean latency of match and mismatch trials.

#### Empathy Quotient

The Empathy Quotient questionnaire (Baron-Cohen & Wheelwright, 2004) comprises 40 empathy questions (e.g., “In a conversation, I tend to focus on my own thoughts rather than on what my listener might be thinking”) and 20 filler questions. Participants were instructed to rate how strongly they agreed or disagreed with each statement (*agree strongly*, *agree slightly*, *disagree slightly*, and *disagree strongly*) without thinking about their responses too much. On each empathy question, participants scored 2 points if the response showed empathy strongly, 1 point if the response showed empathy slightly, or 0 points if the response did not show empathy. The total score was used to indicate participants’ levels of empathy, with a maximum score of 80.

### General Procedure

Participants were tested individually in a quiet room. The testing session lasted approximately 2 hr. Participants read and completed the consent forms, filled out the Empathy Quotient, and then carried out the gesture elicitation tasks. After that, they were given the following cognitive tasks in the same fixed order: the conceptualization task, the picture verification task, the picture naming task, the mental rotation task, the digit span task, the visual pattern task, and the Corsi block task. There were short breaks between the tasks.

In the gesture elicitation tasks, the experimenter faced the participant. The participants’ responses were recorded by a camcorder (PAL DV camera, using 25 frames per second) placed next to the experimenter. In the conceptualization task, the camcorder was placed behind the participant and was used to record his or her verbal description. In all tasks except for the gesture elicitation tasks, participants were instructed to sit on their hands or keep their fingers on the response keys to prevent them from gesturing.

### Coding

#### Speech coding

Verbal responses from the gesture elicitation tasks and the conceptualization task were transcribed verbatim from the video recordings.

#### Gesture classification

Gestures were segmented according to the procedure in Kita, van Gijn, and van der Hulst (1998). They were categorized into four gesture types. The first type of gestures are representational gestures (Kita, 2000), which included depictive and deictic gestures. Depictive gestures can be interpreted in the context of concurrent speech as depicting actions, movements, or perceptual properties of entities. They can also metaphorically depict abstract concepts. Deictic gestures indicate a location or assign meaning to a location in space (McNeill, Cassell, & Levy, 1993). We also counted representational nonhand body part movements as representational gestures (e.g., head tilted to the left to represent one character in the story). The second type of gestures is conduit gestures (McNeill, 1992), which present a clearly formulated idea on the palm to the listener, as if the idea is an object on the open hand. To be coded as a conduit gesture, a gesture needed to fulfill the following criteria: the gesturing hand must move toward the listener; the palm must either face upward or rotate in that direction; at the end of the gesture, the speaker must have eye contact with the listener. This type of gestures is similar to conduit metaphor gestures (McNeill, 1992) and palm presentation gestures (Kendon, 2004) in form and function. These gestures are counted as a subtype of interactive gestures as defined in Bavelas et al. (1992). The third type of gestures is palm-revealing gestures, which express uncertainty, resignation, or show that a speaker has nothing more to say. The hand orientation is typically palm up, or the hand turns to reveal more of the palm. The gesture is often accompanied by a shoulder shrug and/or facial expressions of uncertainty (e.g., an eyebrow raise). The general motivation for the form of these gestures is empty-handedness: The speaker has nothing to show or share. These gestures have very similar functions to the “open hand supine with lateral movement” (Kendon, 2004), but the hand may not always move laterally and the palm may not always face upward. These gestures are counted as a subtype of interactive gestures as defined in Bavelas et al. We also counted a shoulder shrug alone as a palm-revealing gesture if it was used for the same purposes of palm-revealing gestures.^1^ The fourth type of gestures is other gestures, which included beat gestures (simple and rhythmic gestures that do not depict semantic content related to speech), abandoned gestures (gestures that are prematurely stopped before completion), and gestures that could not be coded as the above categories.

#### Gesture saliency

We only coded the saliency of gestures carried out by fingers, hands, or arms, which accounted for 94% of all gestures. Gestures produced by other body parts (e.g., head, shoulder, foot) would require different coding schemes and separate analyses. Saliency was measured on two dimensions: size (the part of hand and/or arm used) and maximum height of the gesturing hand. For size, the part of hand and/or arm was coded into four categories: fingers, hand, forearm, or whole arm, depending on which part of the hand and/or arm was moving. However, if a more proximal part of the hand and/or arm moved slightly as a natural consequence of the movement of the distal part, but the proximal part did not move in an up–down or left–right plane, only the distal part was coded. The height was coded into three categories: below waist, between waist and chin, and above chin. The waist was defined as a straight horizontal line lying between the hips (i.e., the top of a person’s trousers) and the bottom of the rib cage. The chin was defined as a straight horizontal line from the bottom of the chin. For size, a gesture was given 1 point if it was a finger movement and 1 additional point for each increment in size. For height, each gesture was given 1 point if it was below the waist and 1 additional point for each increment in height. The mean score of size and height was used as the indicator of overall gesture saliency. A higher score indicated a more salient gesture.

In order to establish intercoder reliability for the gesture type and saliency coding, the gestures of 24 participants, selected at random (15% of all gestures), were categorized independently by a second coder. Agreement on gesture classification was 92% (Cohen’s κ = .81, *p* < .001). A third coder coded the gesture saliency of the same 24 participants. Agreement on gesture saliency coding with the first coder was 98% (Cohen’s κ = .96, *p* < .001).

### Data Screening

Because correlational analyses and multiple regression analyses are sensitive to extreme outliers, we trimmed the data using the following procedure (in line with the method used in Miyake et al., 2000): For each variable, any observations with values outside 3 standard deviations from the mean were set to values that were 3 standard deviations from the mean. We chose this trimming procedure so that we would not lose participants with extreme values, but these values would not bias the correlations and multiple regressions models. In total, this trimming procedure affected only 0.6% of all observations.

## Results

### Analyses of Gestures

Participants produced a total of 8,646 gestures. Most gestures (67%) were representational gestures, followed by conduit gestures (15%) and palm-revealing gestures (9%). The proportions of nonhand gestures were 5.23% for representational gestures and 28.78% for palm-revealing gestures. All conduit gestures were hand gestures because the palm of the hand had to move toward the listener in order to be coded as a conduit gesture. For each gesture type, gesture frequency was calculated as the number of gestures per 100 words. We collapsed the gesture frequency and saliency data across the two gesture elicitation tasks because gesture frequency and saliency in these two tasks were significantly correlated: gesture frequency, *r*(122) = .76, *p* < .01; gesture saliency, *r*(108) = .62, *p* < .01. We obtained similar results when we analyzed the two tasks separately. Table 1 shows the descriptive statistics for the frequency and saliency of each type of gestures.[Table-anchor tbl1]

### Cognitive and Empathy Variables

Table 2 summarizes the descriptive statistics for the cognitive tasks and Empathy Quotient. Table 3 displays the correlations among the predictor variables. For Table 3, we multiplied latencies and error rates by −1, so that on all predictors higher values indicate better performance. The indicators of working memory capacity (digit span, visual pattern, and Corsi block tasks) were all correlated with each other. Error rates in the digit span task did not correlate with any of the other variables, but error rates of the visual pattern task and Corsi block task positively correlated with the mental rotation response time, the rate of conceptual errors in the conceptualization task, and the latencies of the name–picture verification and picture naming tasks. The rate of conceptual errors in the conceptualization task positively correlated with the latencies on the name–picture verification task and picture naming task, as both tasks involve conceptual processing (but not with the difference score of the two tasks). The name–picture verification latencies positively correlated with the picture naming latencies and with the difference score of the two tasks. Finally, the empathy score did not correlate significantly with any of the other variables.[Table-anchor tbl2][Table-anchor tbl3]

### Predicting Gesture Frequency and Saliency

Table 4 shows the correlations among the dependent variables. The frequency of conduit gestures was positively correlated with the frequencies of both representational and palm-revealing gestures. The frequencies of representational and palm-revealing gestures were not correlated. Finally, the saliency scores of all three types of gestures were highly positively correlated. This suggests that the saliency of gestures may be related to common factors. For the following analyses, we combined the saliency score across all three types of gestures by taking the mean score.[Table-anchor tbl4]

Table 5 shows the correlations between all predictor variables and the dependent variables. The visual and spatial working memory capacity, spatial transformation ability, and conceptualization ability were all negatively correlated with the frequency of representational and conduit gestures. This shows that relatively poor performance on the cognitive tasks was linked to more representational gestures or conduit gestures. Scores on the Empathy Quotient were positively correlated with the frequency of conduit and palm-revealing gestures. This suggests that speakers with higher levels of empathy used these two types of gestures, but not representational gestures, more frequently than speakers with lower levels of empathy.[Table-anchor tbl5]

**Figure 5 fig5:**
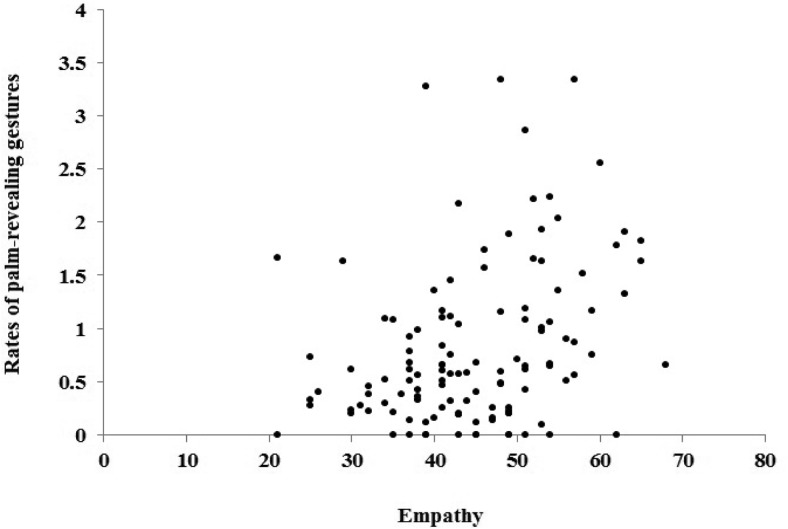
Scatterplot of the correlation coefficient between empathy and frequency of palm-revealing gestures (number of gestures per 100 words).

**Figure 6 fig6:**
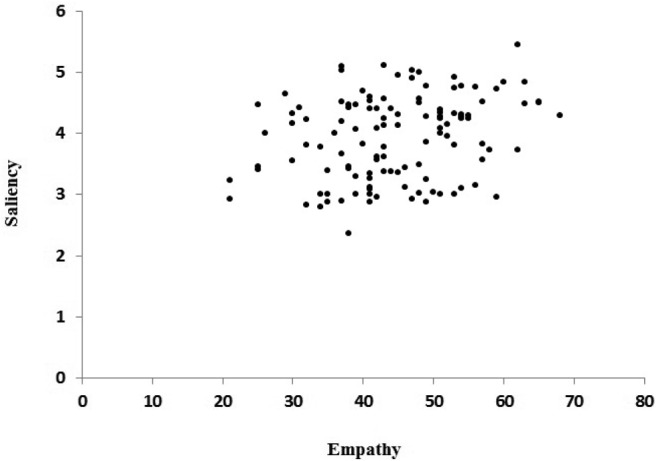
Scatterplot of the correlation coefficient between empathy and scores of gesture saliency.

The overall saliency of gestures was correlated with the empathy scores, but not with the scores in any of the cognitive tasks.^2^ This indicates that participants with higher levels of empathy tended to produce more salient gestures than speakers with lower levels of empathy. Figures 5 and 6 show the scatterplots of the correlation between empathy and the frequency of palm-revealing gestures and the saliency of gestures, respectively.[Fig-anchor fig5][Fig-anchor fig6]

As the frequency of representational and conduit gestures was correlated with more than one predictor variable and some of these predictor variables were correlated with each other, multiple regression analyses were used to determine how well each of the predictor variables independently predicted the frequency of these two types of gestures. As the high positive correlation between visual pattern task performance and Corsi block task performance (*r* = .60) might make it difficult to assess the independent contribution of these two predictor variables, we conducted three multiple regression analyses for the frequency of representational gestures and for the frequency of conduit gestures. The first analysis included only the visual pattern task performance. The second analysis included only the Corsi block task performance. The third analysis included performance on both the visual pattern task and the Corsi block task. Performance on the digit span task, the mental rotation task, the conceptualization task, the lexical retrieval tasks,^3^ and the level of empathy was included in all analyses.

One hundred and twenty-two participants are sufficient for multiple regression analyses with six or seven predictor variables (Green, 1991). In all multiple regression analyses, Cook’s distance, which is a measure of the overall influence of a case on the model, was below 1.00 for all cases. This indicates that no cases had to be excluded as outliers (Cook & Weisberg, 1982). Visual inspection of residuals scatterplots between all predicted dependent variables and errors of predictors indicates that the assumptions of normality, linearity, and homoscedasticity were met in all analyses. The Durbin–Watson test showed that the assumption of independence of errors was met in all analyses (Field, 2009).

For the frequency of representational gestures, all three multiple regression models were significant: with visual pattern task included, *R*^2^ = 22%, *F*(6, 121) = 5.53, *p* < .01; with Corsi block task included, *R*^2^ = 22%, *F*(6, 121) = 5.39, *p* < .01; with both tasks included, *R*^2^ = 23%, *F*(6, 121) = 4.86, *p* < .01. The unique contributions of each predictor are shown in Tables 6, 7, and 8. As the beta weights show in Tables 6 and 7, the frequency of representational gestures was predicted by the score on the visual pattern task, the Corsi block task (marginally significant), the mental rotation task, and the conceptualization task.^4^ This suggests that participants with poorer visual and spatial working memory capacity, spatial transformation ability, or conceptualization ability produced representational gestures more often than those with better abilities. When both predictors were entered into the multiple regression simultaneously, as expected, neither the visual pattern task performance nor the Corsi block task performance was a significant predictor variable due to the fact that the two tasks strongly correlated. Importantly, the mental rotation task performance and the conceptualization task performance were still significant predictors. [Table-anchor tbl6][Table-anchor tbl7][Table-anchor tbl8]

**Figure 7 fig7:**
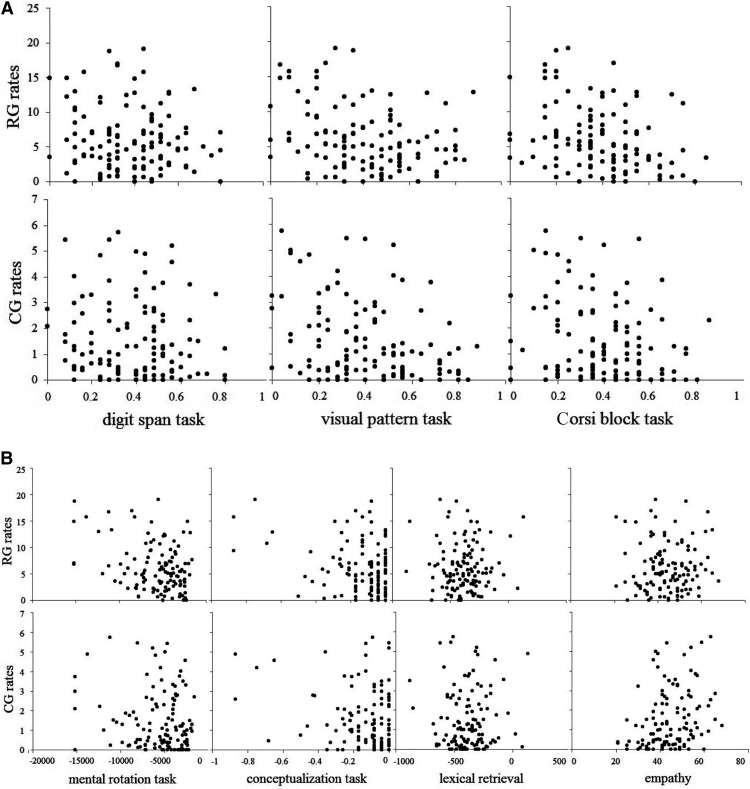
Scatterplot of the correlation matrix between frequencies (calculated as the number of gestures per 100 words) of representational gestures (RG) and conduit gestures (CG) and digit span task, visual pattern task, and Corsi block task (A); and mental rotation task, conceptualization task, lexical retrieval, and empathy (B).

For the frequency of conduit gestures, all three multiple regression models were significant: with visual pattern task included, *R*^2^ = 23%, *F*(6, 121) = 5.76, *p* < .01; with Corsi block task included, *R*^2^ = 17%, *F*(6, 121) = 3.96, *p* < .01; with both tasks included, *R*^2^ = 23%, *F*(6, 121) = 4.95, *p* < .01. The unique contributions of each predictor are shown in Tables 6, 7, and 8. As the beta weights show, the frequency of conduit gestures was predicted by performance on the visual pattern task, the conceptualization task, and the Empathy Quotient score. This suggests that participants’ frequency of conduit gestures was negatively related to their visual working memory capacity or conceptualization ability, and was positively related to their level of empathy. Figure 7 presents the scatterplots of the correlation coefficients between predictor variables and the frequency of representational gestures and conduit gestures.[Fig-anchor fig7]

## Discussion

The aim of the present study was to investigate the relationship between individuals’ cognitive abilities and empathy levels and the frequency and saliency of their gestures. We tested a large heterogeneous sample of speakers from a wide academic spectrum. We focused on the frequency and saliency of three types of gestures: representational gestures, conduit gestures, and palm-revealing gestures. To our knowledge, this is the first study to investigate individual differences in the three main types of gestures within the same speakers and to examine gesture saliency as well as frequency.

Speakers varied substantially in the frequency and saliency of their gestures (see Figures 5–7). To understand why some people gesture more frequently and use more salient gestures than others, we correlated gesture frequency and saliency with indicators of empathy; verbal, visual, and spatial working memory capacity; spatial transformation ability; conceptualization ability; and lexical retrieval ability. We found that levels of empathy predicted the frequency of palm-revealing and conduit gestures, which have communicative functions, and with the saliency of all gestures. Levels of empathy did not predict the frequency of representational gestures. By contrast, spatial and visual working memory capacity, spatial transformation ability, and conceptualization ability predicted the frequency of representational and, to some extent, conduit gestures, but not the saliency of any gestures. Finally, verbal working memory capacity and lexical retrieval ability did not predict the frequency or saliency of any kind of gesture. Below, we comment in more detail on each of these findings.

### Empathy

Speakers with higher levels of empathy, that is, those who are more concerned about others’ understanding and feelings, produced conduit gestures and palm-revealing gestures more often than those with lower levels of empathy. Conduit gestures allow speakers to deliver a clearly formulated idea to their listener and ensure the receipt of the information by making eye contact with their listener. Palm-revealing gestures allow speakers to express their uncertainty or resignation to their listener or to show that they have nothing more to say. Both types of gestures are produced to improve the interaction with the listener (Bavelas et al., 1992). Our findings are consistent with previous evidence that participants produced interactive gestures (which include conduit gestures and palm-revealing gestures) more frequently when they were in face-to-face conversation than when they spoke in a monologue, while the frequency of topic gestures (gestures used to depict semantic information of the concurrent speech, which include representational gestures) did not differ across the two conditions (Bavelas et al., 1992).

Why did empathy not predict the frequency of representational gestures? It may seem plausible that people who consider others’ feelings and understanding more should produce more representational gestures in order to improve the listener’s understanding of the information being expressed. However, this only holds if, in a given task and in the speaker’s opinion, gesturing more frequently indeed improves comprehension. A recent meta-analysis (Hostetter, 2011) examining whether representational gestures improved the listener’s comprehension yielded mixed results. Some studies found that gesture improved listeners’ comprehension, whereas other studies did not find this. Furthermore, listeners benefited more from representational gestures when these gestures were used to depict motor actions than when they were used to depict abstract topics, suggesting that representational gestures are only beneficial for the listener in certain contexts. Additionally, the meta-analysis showed that children benefit more from gestures than adults. As we asked adult participants to describe abstract and social topics, representational gestures might not have contributed significantly to the listener’s comprehension. If the speakers were aware of this, they would not produce representational gestures in order to support communication, even if they had high levels of empathy.

Empathy was the only variable that predicted the saliency of gestures. Speakers with higher levels of empathy produced all three types of gestures more saliently than speakers with lower levels of empathy. By producing more salient gestures, speakers perhaps increase the chance that the listener will understand the information expressed in the gesture. This result is consistent with earlier findings that speakers produce larger gestures when the communicative motivation is stronger than when the communicative motivation is weaker (Holler & Stevens, 2007; Hostetter et al., 2011). The finding that empathy predicted the saliency but not frequency of representational gestures may indicate that the decision on whether to produce a representational gesture might depend primarily on the speaker’s needs to facilitate his or her own speech production, whereas the saliency of a representational gesture might be determined by communicative considerations.

It is worth noting that the present study only used level of empathy as a measurement of how much people care about the clarity of their communication. Future studies could create more direct measurements of how much a person cares about the clarity of communication and correlate these measurements with the frequency and saliency of gestures.

### Visual and Spatial Working Memory Capacity

Participants with poorer visual working memory capacity produced representational gestures and conduit gestures more frequently than participants with better visual working memory capacity. The gesture elicitation tasks in the present study did not require participants to describe visuospatial content, but they still produced representational gestures that metaphorically depicted abstract concepts through movements and locations in space (McNeill, 1992). For instance, when defining *to intervene*, a participant moved the hand with the palm facing downward from shoulder height to stomach height. This gesture expressed the concept of intervention as a physical movement of the hand. These results are consistent with previous findings that speakers produced representational gestures more often when they had to describe pictures from memory than when they could view the pictures during the description (de Ruiter, 1998; Wesp et al., 2001). Furthermore, producing conduit gestures might help speakers to offload mental images to the gesturing hand and therefore increase the activation level of these mental images in visual working memory.

Spatial working memory capacity was negatively related with the frequency of representational gestures. However, this result was only marginally significant and therefore needs to be interpreted with caution. It might suggest that people who have difficulty in memorizing sequences or movements in space tend to produce representational gestures to help them remember these sequences or movements. Spatial working memory capacity did not predict the frequency of conduit gestures. This is a null result and so is difficult to interpret. One might speculate, however, that the results for the two gesture types differ because complex sequences of positions and movements, assessed in the spatial working memory task, are more likely to be expressed in representational than in conduit gestures.

### Spatial Transformation and Conceptualization Abilities

As explained in the introduction, representational gestures may support the generation of the conceptual contents of utterances in two ways, namely, by supporting the transformation of mental representations and by supporting the segmentation of messages into units suitable for expression (Alibali et al., 2000; Kita, 2000). Our findings are in line with this view. First, participants who performed worse in the mental rotation task, that is, those who found it more difficult to transform mental images, produced representational gestures more frequently than those who performed better. Representational gestures might be used to simulate the transformation of mental images, which could provide vivid visual and proprioceptive representations and consequently facilitate mental transformation. This result is consistent with previous findings that representational gestures and spatial transformation are closely linked: People produce more representational gestures when spatial transformation is more difficult than when it is easier (Chu & Kita, 2011; Hostetter et al., 2007); and encouraging the use of representational gestures improves performance in mental rotation tasks, compared to prohibiting the use of representational gestures (Chu & Kita, 2011).

Second, participants who performed worse on the conceptualization task produced representational gestures more frequently than those who performed better. The same held for conduit gestures. In the conceptualization task, participants were asked to describe a set of lines, which were sometimes superimposed with dark lines that created distracting shapes. To perform well on this task, one has to suppress irrelevant information and segment the relevant information into suitable units for speaking. Both representational gestures and conduit gestures may facilitate focusing on the relevant information and/or reduce distraction from irrelevant information. Our results are consistent with previous results showing that adults and children produced representational gestures more often when they described conceptually more difficult diagrams than when they described less difficult ones (Alibali et al., 2000; Hostetter et al., 2007; Kita & Davies, 2009; Melinger & Kita, 2007).

In sum, our findings are consistent with the view that representational gestures support the generation of the conceptual contents of speech in two ways: by supporting the transformation of mental imagery and by facilitating the segmentation of messages into units suitable for expression.

### Verbal Working Memory Capacity and Lexical Retrieval Ability

Our results failed to show any significant relationship between the participants’ verbal working memory capacity or lexical retrieval ability and the frequency or saliency of any type of gesture. These findings are null results and need to be interpreted with caution. It is possible that verbal working memory was not strongly implicated in the gesture elicitation tasks used in the present study. The participants provided definitions of relatively common phrases and discussed a social dilemma without any constraints on the content of their speech. Thus, the load on verbal working memory may have been low. In order to assess whether the speakers’ verbal working memory capacity is related to his or her use of representational gestures, it may be necessary to use elicitation tasks that force speakers to retain verbal information in verbal working memory. Similarly, the current gesture elicitation tasks may not have been particularly taxing with respect to lexical retrieval, as speakers were entirely free in their choice of words. In future research one might aim to constrain the participants’ choice of words in order to increase the difficulty of lexical access.

### Suggestions for Future Research

We showed that the predictors used in the current study accounted for some, but by no means all, of the variance in the speakers’ gesture frequency and salience. Clearly, much more work needs to be done in order to understand why speakers in all cultures use gesture but differ substantially in when and how they do so. Here we highlight four possible extensions of our work.

First, within the current project we could only assess each trait through a single task. Though these tasks were carefully selected to capture the target traits, they may have captured other traits as well. This “task impurity” problem can be alleviated by assessing each trait through several tasks, which, apart from the target trait, share little variance (e.g., Miyake & Friedman, 2012; Miyake et al., 2000). Statistical analyses can then be used to extract the underlying latent variables and use these, rather than the scores on the individual tasks, to predict the target behavior. This approach has been used in various areas of individual differences research, including studies concerning leadership (e.g., Chan & Drasgow, 2001), printed word recognition (e.g., Gayán & Olson, 2003), and working memory (e.g., Bayliss, Jarrold, Gunn, & Baddeley, 2003). Such an approach would be highly beneficial in research on individual differences in gesture use as well.

Second, gestures could be elicited in a wider range of tasks than used here. The elicitation tasks should be selected such that the importance of specific predictors can be optimally assessed. As suggested above, in order to assess the importance of verbal working memory capacity and lexical retrieval ability as predictors of gesture frequency and saliency, one might use the tasks where the maintenance of verbal information and the lexical retrieval are more challenging than they were in the present study. By systematically varying specific features of the gesture elicitation tasks (for instance, the difficulty of selecting words), a better understanding can be reached of how task constraints and speaker traits jointly determine the use of gestures.

Third, future studies could test more diverse groups of speakers than the present study. We assessed speakers varying widely in educational background, which served our purposes better than assessing only university students, as is common practice in psycholinguistics. Whether our results, obtained with a sample of young people from urban Britain, would generalize to older speakers or speakers with different cultural backgrounds remains to be established. The choice of participants should be guided by hypotheses about the impact different predictors may have in specific groups.

Fourth, correlational research can uncover relationships between gestures and cognitive variables. Yet, in order to understand why they exist, experimental research is essential. In such research, it is important to distinguish research questions concerning the origins or causes of gestures from questions about their functions or effects. The origins of gesture can be investigated by, for instance, manipulating the difficulty of a particular cognitive operation (say, mental rotation) in a speech production task and observing the effect on gesture frequency (e.g., de Ruiter, 1998; Hostetter et al., 2007; Kita & Davies, 2009; Melinger & Kita, 2007; Wesp et al., 2001). The functions of gestures may be studied by comparing the performance in a linguistic task when gesturing is allowed (or encouraged) to when it is prohibited (e.g., Chu & Kita, 2008; Frick-Horbury & Guttentag, 1998; Mol & Kita, 2012; Rauscher et al., 1996).

### Conclusion

The present study measured the associations between indicators of empathy and several cognitive skills and the frequency and saliency of three types of gestures, namely, palm-revealing, conduit, and representational gestures. We found that empathy predicted the saliency of all three types of gestures and the frequency of gestures with interactive function, that is, conduit and palm-revealing gestures, but not the frequency of representational gestures. Conversely, individuals’ cognitive abilities (e.g., visual and spatial working memory capacity, spatial transformation ability, conceptualization ability) were related to the frequency of representational and conduit gestures, but none of them predicted the saliency of any type of gesture. Our results indicate that a broad correlational approach, simultaneously considering different types of gestures and a wide range of predictors, is likely to be fruitful in further work aiming to understand individual differences in gesture use.

## Supplementary Material

10.1037/a0033861.supp

## Figures and Tables

**Table 1 tbl1:** Descriptive Statistics for the Dependent Variables

Variable	*M*	*SD*	Minimum	Maximum	*N*
Frequency					
Representational gestures	6.05	4.48	0.00	19.07	122
Conduit gestures	1.47	1.48	0.00	5.74	122
Palm-revealing gestures	0.80	0.75	0.00	3.34	122
Saliency					
Representational gestures	4.01	0.74	2.34	5.65	114
Conduit gestures	3.82	0.71	2.42	5.50	107
Palm-revealing gestures	3.67	0.71	2.00	5.50	100
*Note*. Frequencies were calculated as the number of gestures per 100 words. Saliency data only include gestures produced by fingers, hands, or arms, and nonhand gestures were excluded from saliency analyses.					

**Table 2 tbl2:** Descriptive Statistics for the Predictor Variables (*N* = 122)

Measure	*M*	*SD*	Minimum	Maximum
Digit span task				
Proportion of correct recalls	0.38	0.18	0.00	0.80
Visual pattern task				
Proportion of correct recalls	0.41	0.21	0.00	0.88
Corsi block task				
Proportion of correct recalls	0.39	0.18	0.00	0.85
Mental rotation task				
Error rates	0.33	0.16	0.04	0.67
Reaction time (ms)	5,668	3,084	1,660	15,230
Conceptualization task				
Number of errors per box	0.14	0.17	0.00	0.87
Picture naming task				
Error rates	0.19	0.10	0.02	0.47
Reaction time (ms)	919	153	632	1,437
Name–picture verification task				
Error rates	0.07	0.04	0.01	0.35
Reaction time (ms)	536	94	380	960
Naming task–Verification task				
Reaction time (ms)	383	161	−13	880
Empathy Quotient				
Total empathy score out of 80	44.66	9.82	21.00	68.00

**Table 3 tbl3:** Pearson Correlation Coefficients for the Predicator and Dependent Variables (*N* = 122)

Variable	1	2	3	4	5	6	7	8	9
1. Digit span task	—								
2. Visual pattern task	.32**	—							
3. Corsi block task	.30**	.60**	—						
4. Mental rotation task	.05	.19*	.24**	—					
5. Conceptualization task	−.02	.27**	.21*	.10	—				
6. Picture verification task	.02	.30**	.31**	.17	.26**	—			
7. Picture naming task	.03	.15	.15	.26**	.11	.22**	—		
8. Naming task–Verification task	.02	−.04	−.04	.14	.05	−.38**	.82**	—	
9. Empathy Quotient	−.04	−.01	−.07	−.11	.06	.05	.01	−.03	—
*Note*. The negative values of reaction time or error rate were used for the mental rotation task, name–picture verification task, picture naming task, and conceptualization task so that higher scores always indicate better performance.									
* *p* < .05. ** *p* < .01.									

**Table 4 tbl4:** Pearson Correlation Coefficients for the Dependent Variables (*N* = 122 Unless Noted)

Variable	1	2	3	4	5	6
1. Frequency of representational gestures	—					
2. Frequency of conduit gestures	.48**	—				
3. Frequency of palm-revealing gestures	.18*	.19*	—			
4. Saliency of representational gestures^a^	.41**	.18^a^	.18^†^	—		
5. Saliency of conduit gestures^b^	.37**	.15	.16	.81**	—	
6. Saliency of palm-revealing gestures^c^	.37**	.07	.09	.72**	.56**	—
*Note*. Frequencies were calculated as the number of gestures per 100 words. Saliency data only include gestures produced by fingers, hands, or arms, and nonhand gestures were excluded from saliency analyses.						
^a^ *N* = 114. ^b^ *N* = 107. ^c^ *N* = 100.						
^†^ *p* = .06. * *p* < .05. ** *p* < .01.						

**Table 5 tbl5:** Pearson Correlation Coefficients for the Predicator and Dependent Variables (*N* = 122 Unless Noted)

Predictor	Gesture frequency			Gesture saliency (all)^a^
	Representational	Conduit	Palm revealing	
Digit span task	−.12	−.08	−.04	−.08
Visual pattern task	−.31**	−.35**	−.02	−.13
Corsi block task	−.30**	−.21*	−.10	−.08
Mental rotation task	−.34**	−.16	−.13	−.17
Conceptualization task	−.29**	−.26**	.01	−.13
Naming task reaction–Verification task reaction time	−.01	.01	.01	−.02
Empathy Quotient	.02	.27**	.33**	.28**
*Note*. Frequencies were calculated as the number of gestures per 100 words. Saliency data only include gestures produced by fingers, hands, or arms, and nonhand gestures were excluded from saliency analyses. The negative values of reaction time or error rate were used for the mental rotation task, name–picture verification task, picture naming task, and conceptualization task so that higher scores always indicate better performance.				
^a^ *N =* 118.				
* *p* < .05. ** *p* < .01.				

**Table 6 tbl6:** Results of Multiple Regression Analyses, With the Visual Pattern Task (But Not the Corsi Block Task) Included in the Predictors

Predictor	Frequency of representational gestures		Frequency of conduit gestures	
	β	*t*	β	*t*
Digit span task	−.05	−0.62	.02	0.27
Visual pattern task	−.18	−1.97*	−.29	−3.21**
Mental rotation task	−.29	−3.43**	−.05	−0.62
Conceptualization task	−.21	−2.44*	−.19	−2.25*
Naming task–Verification task	.04	0.47	.00	0.03
Empathy Quotient	−.01	−0.06	.27	3.24**
*Note*. Frequencies were calculated as the number of gestures per 100 words. The negative values of reaction time or error rate were used for the mental rotation task, name–picture verification task, picture naming task, and conceptualization task so that higher scores always indicate better performance.				
* *p* < .05. ** *p* < .01.				

**Table 7 tbl7:** Results of Multiple Regression Analyses, With the Corsi Block Task (But Not the Visual Pattern Task) Included in the Predictors

Predictor	Frequency of representational gestures		Frequency of conduit gestures	
	β	*t*	β	*t*
Digit span task	−.07	−0.75	−.04	−0.45
Corsi block task	−.16	−1.79^†^	−.11	−1.12
Mental rotation task	−.29	−3.30**	−.08	−0.85
Conceptualization task	−.23	−2.65*	−.25	−2.82*
Naming task–Verification task	.04	0.44	.01	0.13
Empathy Quotient	−.01	−0.16	.26	3.05**
*Note*. Frequencies were calculated as the number of gestures per 100 words. The negative values of reaction time or error rate were used for the mental rotation task, name–picture verification task, picture naming task, and conceptualization task so that higher scores always indicate better performance.				
^†^ *p* = .08. * *p* < .05. ** *p* < .01.				

**Table 8 tbl8:** Results of Multiple Regression Analyses, With Both Visual Pattern Task and Corsi Block Task Included in the Predictors

Predictor	Frequency of representational gestures		Frequency of conduit gestures	
	β	*t*	β	*t*
Digit span task	−.04	−0.48	.02	0.19
Visual pattern task	−.13	−1.22	−.32	−3.04**
Corsi block task	−.10	−0.92	.06	0.55
Mental rotation task	−.28	−3.23**	−.06	−0.70
Conceptualization task	−.20	−2.36*	−.20	−2.27*
Naming task–Verification task	.04	0.42	.01	0.06
Empathy Quotient	−.01	−0.12	.27	3.26**
*Note*. Frequencies were calculated as the number of gestures per 100 words. The negative values of reaction time or error rate were used for the mental rotation task, name–picture verification task, picture naming task, and conceptualization task so that higher scores always indicate better performance.				
* *p* < .05. ** *p* < .01.				

## Footnotes

1 Shoulder shrugs were also coded as palm-revealing gestures because we believe they serve the same function of palm-revealing gestures.

2 For all types of gesture, the size score was positively correlated with the height score, *r*(116) = .63, *p* < .001. The level of empathy was positively correlated with the size score, *r*(116) = .32, *p* < .001, and was marginally correlated with the height score, *r*(116) = .15, *p* = .10.

3 We also assessed the lexical retrieval efficiency in a different way by computing residuals obtained by regressing the name–picture verification latencies from picture naming latencies (see DeGutis, Wilmer, Mercado, & Cohan, 2013). Participants’ residuals and subtraction scores were highly correlated, *r*(122) = .90, *p* < .001, and consequently, replacing the subtraction score with the residuals did not change any relevant results reported in this article. We thank Jeremy Wilmer for suggesting the residual-based analyses to us.

4 There was no speed–accuracy trade-off between the reaction time and the error rate in the mental rotation task, *r*(122) = −.02, *p* = .82. We obtained essentially the same results for all analyses when the error rate of the mental rotation task was used as an indicator of spatial transformation ability instead of reaction time. Most importantly, when error rate was entered into the multiple regression analysis, it was a significant predictor of the rates of representational gestures (*p* = .05).
