# Supplementary material for: Individual Differences in Frequency and Saliency of Speech-Accompanying Gestures: The Role of Cognitive Abilities and Empathy
Source: J Exp Psychol Gen. 2013 Aug 5;143(2):694–709. doi: 10.1037/a0033861 (PMC3970852; doi:10.1037/a0033861)
Supplement: Supplementary file 1 [file Gesture_coding_manual.pdf]

# GESTURE CODING MANUAL

## GESTURE TYPE CLASSIFICATION

### *1. Representational gesture*

Representational gestures depict semantic content via the shape, placement, and/or motion trajectory of the hands. Representational gestures include depictive (i.e., iconic/metaphorical) and deictic gestures. Depictive gestures can be interpreted, in the context of concurrent speech, as representing a real or metaphorical action (e.g., a speaker moves two hands towards each other while saying, “bring my friends together”) or depicting properties of a real or metaphorical referent (e.g., a speaker moves his hand from left to right while saying, “when something changes”). Deictic gestures indicate a location of a real or metaphorical referent (e.g., a speaker points to the left to refer to “Amy” and then points to the right to refer to “Peter”).

Sometimes a representational gesture is performed by a non-hand body part. For example, a speaker moves her head to the left to refer to “Amy” and then to the right to refer to “Peter”.

This gesture type includes iconic gestures, metaphorical gestures and deictic gestures in McNeill's (1992) classification (As for deictic gestures, in our data set we would only see "abstract deictic gestures" without a physically present target of pointing).

### *2. Conduit gesture*

Conduit gestures can be interpreted as a speaker presenting an idea or concept on his/her palm to the listener. The speaker must have eye contact with the listener at the end of the gesture. The speaker’s hand must move towards the listener (not down or sideways), and their palm must be revealed to the listener.

When producing a conduit gesture, speakers often intonate their speech as if to emphasize the idea or concept that they want to express to the listener. The speaker may also show other nonverbal cues that suggest orientation towards the listener such as leaning forward (towards the listener).

Conduit gestures are always performed by the hand.

The term and concept of conduit gestures are taken from McNeill (1992) ("conduit metaphor gesture"). It is similar to Kendon's (2004) "palm presentation gestures". It is a subtype of interactive gestures in Bavelas, Chovil, Lawrie, and Wade (1992).

### *3. Palm-revealing gesture*

Palm-revealing gestures can be interpreted as a speaker using an empty hand to show that he/she has nothing to show to or share with the listener. The hand orientation is typically palm up, or the hand turns to reveal the palm. It is often accompanied by a shoulder shrug and/or facial expressions (e.g., eyebrow raises and lips tighten and turn down at the ends).

Palm-revealing gestures occur in the following three situations:

1) A speaker is uncertain about what she is talking about. The uncertainty is indicated by indecision or vagueness in the concurrent speech (e.g., “it’s like”, “kind of”, “I don’t know”) or speech disfluency (e.g., stuttering, repetition, filled pauses or silent pauses).
2) A speaker has nothing more to say. The palm-revealing gesture is typically accompanied by speech such as, "that's all", "that's it", or a silent period at the end of a description.
3) The speaker expresses resignation without any speech, as though to say, “I wouldn’t have a choice” or “That’s just how it is”.

A shoulder shrug (without any hand movement) is coded as a palm-revealing gesture if used for the same purposes described above

The critical difference between a conduit gesture and palm-revealing gesture is that the former is produced to express a clear message to the listener, while the latter is produced to show that the speaker is uncertain, resignation, or to show one has nothing more to say.

It is a subtype of interactive gestures in Bavelas et al. (1992). It has very similar functions to Kendon's (2004) “Open Hand Supine with lateral movement”, but the hand does not always move laterally and the palm is not always facing upwards.

***4. Other types of gesture***

**4.1 Beat gesture**

Beat gestures are motorically simple, rhythmic gestures that do not depict semantic content related to speech. A beat gesture has two movement phases (i.e., up and down). There may be several up-and-down movements in succession. The hand shape is open and lax. The gesture happens in the periphery of the gesture space (e.g. on the lap). The term and concepts are from McNeill (1992).

**4.2 Abandoned gesture**

These are the gestures that are prematurely stopped before completion. They only consist of a preparation and a retraction phase, without any stroke phase.

**4.3 Unclear gesture**

These are gestures that cannot be coded as any of the categories above.

## GESTURE SALIENCY CLASSIFICATION

### SIZE CODING

Gesture size is categorized into the following four categories:

1. Finger(s) only: The finger(s) move, but the hand remains still
2. Hand: The hand(s) move at the wrist in an up/down or left/right plane, but the forearm remains still. Or the hand rotates and the forearm rotates in consequence*.

3. Forearm: The forearm(s) move in an up/down or left/right plane, but the upper arm remains still. If the forearm rotates only as a result of the hand rotating, this is coded as hand only*.
4. Whole arm: The whole arm, including the upper arm moves in an up/down or left/right plane.

*It is worth noting that when one body part moves, a bigger, connecting body part may move slightly as a natural consequence. If the bigger part moves only slightly, and not in an up/down or left/right plane, the smaller body part will be coded as the gesture size. However, if the bigger body part moves in an up/down or left/right plane, and is more than a natural consequence of the smaller body part moving, the bigger part should be coded.

## HEIGHT CODING

Height coding is based on the highest point of the hand/finger (whichever is highest) in the gesture. Gesture height is categorized into the following three categories:

1. lower than waist
2. between waist and chin
3. above chin

The waist was defined as a horizontal line midway between the speaker's hips and the bottom of his/her ribcage. The chin was defined as a horizontal line from the bottom of the speaker's chin.

## **REFERNCES**

Bavelas, J. B., Chovil, N., Lawrie, D. A., & Wade, A. (1992). Interactive gestures. *Discourse Processes, 15,* 469-489.

Kendon, A. (2004). *Gesture: Visible action as utterance.* Cambridge, UK: Cambridge University Press.

McNeill, D. (1992). *Hand and Mind.* Chicago: University of Chicago Press.
